# Dissociative experiences among Lebanese university students: Association with mental health issues, the economic crisis, the COVID-19 pandemic, and the Beirut port explosion

**DOI:** 10.1371/journal.pone.0277883

**Published:** 2022-11-18

**Authors:** Mariam Mhanna, Christian-Joseph El Zouki, Abdallah Chahine, Sahar Obeid, Souheil Hallit

**Affiliations:** 1 School of Medicine and Medical Sciences, Holy Spirit University of Kaslik, Jounieh, Lebanon; 2 Social and Education Sciences Department, School of Arts and Sciences, Lebanese American University, Jbeil, Lebanon; 3 Research Department, Psychiatric Hospital of the Cross, Jal Eddib, Lebanon; 4 Applied Science Research Center, Applied Science Private University, Amman, Jordan; Chiang Mai University, THAILAND

## Abstract

**Background:**

Dissociative experiences are psychological manifestations characterized by a loss of connection and continuity between thoughts, emotions, environment, behavior, and identity. Lebanon has been facing indescribable events in the last few years, including the COVID-19 pandemic, the Beirut explosion, a crushing economic crisis with the highest inflation rate the country has known in over three decades. The aim of this study was to evaluate the correlation between dissociative experiences and post-traumatic stress symptoms from the economic crisis, the Beirut blast, the COVID-19 pandemic, and other mental health issues in a sample of Lebanese university students.

**Methods:**

This cross-sectional study enrolled 419 active university students (18–35 years) from all over Lebanon (May and August 2021). The respondents received the online soft copy of a survey by a snowball sampling technique through social media and messaging apps. The questionnaire included sociodemographic data, the Dissociative Experience Scale (DES-II), the PTSD Checklist Specific Version (PCL-S), the Financial Wellbeing Scale, the Beirut Distress Scale, the Lebanese Anxiety Scale, the Patient Health Questionnaire.

**Results:**

The two-factor model of the DES fitted best according to CFI, RMSEA and χ^2^/df values, but modestly according to TLI. The two factors were absorption and amnesia/depersonalization. Higher stress (Beta = 0.95) and more PTSD from the Beirut blast (Beta = 0.29) and from the economic crisis (Beta = 0.23) were significantly associated with more absorption. A personal history of depression (Beta = 6.03), higher stress (Beta = 0.36) and more PTSD from the Beirut blast (Beta = 0.27) and from the COVID-19 pandemic (Beta = 0.16) were significantly associated with more amnesia/depersonalization.

**Conclusion:**

Significant rates of dissociative experiences and their sub-manifestations (amnesia/depersonalization and absorption) were found among Lebanese university students, with remarkable co-occurrence of a traumatic/stressful pattern, whether on an individual (history of PTSD) or a collective level (Post-traumatic manifestations from Beirut blast, COVID-19 pandemic and/or economic crisis), or whether correlated to an acute single event or to certain chronic stressors, or even to a personal history of depression. Such findings must raise the attention to serious mental and psychosocial alteration in the Lebanese national identity.

## Introduction

Dissociative experiences are cognitive processes that manifest itself in a multitude of symptoms. The Diagnostic and Statistical Manual of Mental Disorders (DSM-5) determined many features linked to dissociation such as a disruption of and/or discontinuity in the normal integration of consciousness, memory, identity, emotion, perception, body representation, motor control, and behavior [1]. Dissociation can be a very complex experience to describe, as the person going through it may disconnect from the rest of the world and have the impression that the world around him is not real. People suffering from dissociative symptoms can also show signs of amnesia, derealization/depersonalization, and absorption [2]: First, dissociative amnesia is a failure to recall significant autobiographical information, frequently of a traumatic or distressing origin, that is not explained by normal forgetting [1]. Second, derealization/depersonalization is the perception of somatic or cognitive detachment from oneself or one’s environment [1]. Third, absorption is another form of dissociative symptoms: It means that people ignore environmental stimuli surrounding them in favor of one stimulus, whether external (like a movie) or internal (like a thought), becoming completely absorbed by it and giving it total attention [3]. A recent meta-analysis reported that 11.4% of college students meet criteria for dissociative disorders [4].

Dissociation has been demonstrated to have a wide range of repercussions, as it has the capacity to disrupt every area of mental functioning, including the development of suicidal ideas and non-suicidal self-injury in advanced cases [5]. Characteristics of dissociation can be seen in non-clinical samples but at low scores, more pronounced symptoms are common in virtually all mental illnesses, including dissociative disorders, posttraumatic stress disorder, and borderline personality disorder [6]. Dissociative experiences are common following a traumatic event, and this trauma can potentially lead to the development of full-blown dissociative disorders [1].

Dissociative symptoms such as amnesia, flashbacks, numbness, and depersonalization/derealization are common in posttraumatic stress disorder [1]. Dissociative experiences were also correlated to depression in a sample of sexually abuse survivors [7], and it was also proven in a recent study among women suffering from fibromyalgia or rheumatoid arthritis [8]. A prospective investigation found that levels of stress are positively correlated to dissociation as well [9]. Anxiety was also found to be prevalent in dissociation, as the latter is transdiagnostically present in many anxiety disorders [10].

Dissociative symptoms are common following traumatic events and in PTSD. Moreover, it has been proven that PTSD, anxiety, and depression are deeply interconnected. Data shows that some of the most common comorbid conditions with post-traumatic stress following traumatic experiences are anxiety, depression, and stress [11]. A recent study done in the USA found that many young adults suffered from post-traumatic stress, anxiety and depressive symptoms during the COVID-19 pandemic, showing that there is a possibility of co-occurrence and overlaps of much symptomatology, especially in the youth [12]. PTSD can have multiple explanatory factors like economic problems and traumatic events [13]. Other factors that can play a mediating role between PTSD and dissociation are anxiety and depression [14].

Lebanon is a country that is constantly tormented by internal and external conflicts, which provides fertile ground for development of PTSD and dissociative manifestations [15]. A number of studies targeted the evaluation of PTSD prevalence in the Lebanese population showing a range of 15.4% to 35.0% in adolescents for the July War of 2006 [13], and a general elevation in the PTSD rates compared to more peaceful countries [16]. An additional paper also detected significant rates of post-traumatic stress symptoms in a sample of university students in Lebanon [17]. The Lebanese population have endured multiple sufferings in a short period of time. Despite living in an unstable country for almost four decades of war and political unpredictability, they seemed to have the ability to cope with traumas [15]. Nonetheless, since 2019, the Lebanese people faced one of the worst economic crisis in the world since 1850, the heavy load of the COVID-19 pandemic and the Beirut post explosion, all in a period of 12 months [18], which lead to a spike in mental health issues [19]. Since its emergence, the COVID-19 pandemic, has shown to be detrimental on the psychological wellbeing of the individual by swelling the levels of stress, anxiety, somatization, depression, and post-traumatic stress [20, 21]. On August 4th 2020, the biggest non-nuclear explosion in history occurred in Beirut, the capital of Lebanon, aggravating the socio-economic situation in the country and escalating the transmission of COVID-19, leaving the people with a triple strike of trauma [22]. The pandemic and the Beirut port explosion came on top of a crushing economic crisis with the highest inflation rate the country has seen in over thirty years [23]. The Lebanese people are facing several mental issues as demonstrated in the latest body of research conducted in the country [24–33].

Mental illness is becoming highly prevalent in college students [34]. In addition to scholastic stress, students may be faced with the responsibility of taking on more adult-like duties and challenges without having yet acquired the skills and cognitive maturity of adulthood [35]. A study published in the Journal of Nervous and Mental Disease showed that dissociative experiences scores are significantly stable over time, thus it can be stipulated that people suffering from dissociative manifestations are very likely to carry the burden of those complex symptoms for a long period of time [36].

As a result, we determined that assessing the severity of dissociation following traumatic events in a group of college students in Lebanon was critical knowing that, to the best of our knowledge, there is a lack of evidence about the severity of dissociative experiences (total dissociation score, dissociative amnesia, absorption, depersonalization/derealization) and their correlation with PTSD, stress, anxiety, and depression among university students, particularly in the Lebanese setting. In addition, this study might be one of the first to (1) assess PTSD-like symptoms that is directly caused by an economic crisis, and to (2) evaluate the repercussions of the Beirut Blast on university students.

Based on a survey of college students, we evaluated the intensity of dissociative experiences and other mental manifestations, as well as their relationship with multiple traumatic occurrences. We initially hypothesized that dissociation levels will be elevated in our sample, and that it will be correlated with higher levels of post-traumatic stress (from the Beirut Blast, the COVID-19 Pandemic, and the Economic Crisis) and more pronounced stress, anxiety, and depression.

Thus, the aim of this study was to evaluate the correlation between some dissociative experiences and PTSD from economic crisis, Beirut blast, and the COVID-19 pandemic. An additional objective was to also find an association between dissociation and other mental health issues such as anxiety, stress, and depression. The findings of this research can be used as a foundation for future studies on PTSD, dissociative experiences, and their correlates. This study can provide more insight to the scientific dialogue in Lebanon regarding mental health issues by filling in the gaps in current knowledge.

## Methods

### Ethics approval and consent to participate

The Psychiatric Hospital of the Cross Ethics and Research Committee approved this study protocol (HPC-028-2021). An informed written consent was considered obtained from each participant when submitting the online form. All methods were performed in accordance with the relevant guidelines and regulations.

### Study design

This cross-sectional study was conducted between May and August 2021. It was filled by 419 university students (124 males, 295 females) from all Lebanese governorates. We did not define any specific inclusion criteria for potential participants during the recruitment process, but we requested that all respondents should be university students and that resided in Lebanon during the COVID-19 pandemic, the Beirut port explosion, and the Economic Crisis. Snowball sampling and respondent-driven sampling techniques were implemented for data collection and was this due to the challenging pandemic imposing social distancing, and the closure of most of the universities in the country. To reduce bias due to our convenience sampling method, we worked on diversifying our data collection by enlisting as many participants as possible from various backgrounds and locations. Consequently, a soft copy of the questionnaire was created using Google Forms and sent to participants via universities’ internal emails, social media platforms and messaging applications. Prior to participation, study objectives and general instructions were thoroughly explained. No credits were received for participation.

### Minimal sample size calculation

As a rule of thumb, simulation studies show that with normally distributed indicator variables and no missing data, a reasonable sample size for a simple confirmatory factor analysis model is about N = 150 [37]. For the remaining analysis, the G-power software calculated a minimal sample of 395 students, based on an effect size f2 = 2%, α error = 5%, power = 80%, and a maximum of 15 factors to be included in the multivariable analysis.

### Questionnaire

The survey was conceived in English, as it is widely spoken in the country and one of the most frequently used and assimilated languages in Lebanese universities. The first part of the questionnaire was a consent form, where respondents gave their approval to fill the questionnaire. The second part constitutes of questions assessing socio-demographic details (age, university, height, weight, etc.). The household crowding index (HCI), reflecting the socioeconomic status (SES) of the family, was calculated by dividing the number of persons living in the house by the number of rooms in the house; higher HCI reflect a lower SES. The third part comprised the following measures:

#### Dissociative Experience Scale (DES)

This tool is a 28-item self-reported questionnaire addressing the frequency of certain dissociative experiences of varying severity, such as derealization, amnesia, depersonalization, absorption, gaps in awareness, and imaginative involvement. We calculated the total score by averaging the 28 items’ scores [38], with higher scores indicating higher dissociative experience.

#### The PTSD Checklist- Specific Version (PCL-S)

It is a 17-item self-report questionnaire in which each item corresponds to symptoms of PTSD. Items are rated on a Likert scale ranging from 1 (not at all bothersome) to 5 (extremely bothersome) [39]. Of the three versions of the PCL, we used for the current study the “specific” version, in which the management of the index trauma sets symptom ratings to a certain event, such as the Lebanese economic crisis, the COVID-19 pandemic and the Beirut blast event (current Cronbach’s α = 0.945 for COVID-19 pandemic, 0.947 for the Beirut blast and 0.959 for the economic crisis).

#### Financial wellbeing scale

It is an 8-item scale rated from 1 to 10, with 10 being the least pressure felt from the current financial situation in the country [40] (current Cronbach’s α = 0.931). Written permission was obtained to use this copyrighted scale.

#### Beirut Distress Scale (BDS‐10)

It is a 10-item scale to evaluate psychological and mental distress. The items are rated on a Likert scale from 0 (never) to 3 (very much), with higher scores showing higher stress levels [41] (current Cronbach’s α = 0.886).

#### Lebanese Anxiety Scale (LAS)

It’s a 10-item instrument, with seven questions rated from one to four and three questions graded from 0 to 3; higher scores indicate more anxiety [31, 42] (current Cronbach’s α = 0.907).

#### The Patient Health Questionnaire-9 (PHQ-9)

Developed by Spitzer et al in 2001 [43] and validated in Lebanon [44], the PHQ-9 is a self-administered scale that assesses depressive symptoms, with questions rated from “0” (not at all) to “3” (nearly every day), in the 14 days prior to evaluation (current Cronbach’s α = 0.887).

### Data analysis

The SPSS AMOS software v.24 was used to conduct confirmatory factor analyses. Multiple indices of goodness-of-fit were described: the Relative Chi-square (χ2/df) (cut-off values:<2–5), the Root Mean Square Error of Approximation (RMSEA) (close and acceptable fit are considered for values <0.05 and <0.11 respectively), the Tucker Lewis Index (TLI) and the Comparative Fit Index (CFI) (acceptable values are ≥0.90) [45, 46].

Statistical analysis was performed using SPSS software, version 23. The dissociative experiences score had a normal distribution since the skewness and kurtosis values varied between -1 and +1 [47], with a sample larger than 300 [48]. Accordingly, the Student t-test was used to check for an association between the dissociative experiences score and dichotomous variables (i.e., gender) while the Pearson correlation test was used to correlate two continuous variables (i.e., age, BMI). A stepwise linear regression was conducted taking the dissociative experiences score as the dependent variable. Covariates that were included in the linear regression model were those that showed an effect size or correlation > │0.24│ in the bivariate analysis to have parsimonious models [49]. P<0.05 was considered significant.

## Results

The sample consisted of 419 participants, with a mean age of 21.02 ± 2.59 years and 70.4% females. Other characteristics and description of the scores can be found in Table 1.

**Table 1:** Sociodemographic characteristics of the participants (N = 419).

Variable	N (%)
Gender	
Male	124 (29.6%)
Female	295 (70.4%)
Personal history of anxiety disorders (yes)	83 (19.8%)
Personal history of PTSD (yes)	26 (6.2%)
Personal history of depression (yes)	82 (19.6%)
	**Mean ± SD**
Age (in years)	21.02 ± 2.59
Household crowding index	0.91 ± 0.42
Financial wellbeing scale	39.19 ± 16.61
Depression (PHQ-9 score)	11.24 ± 6.60
Anxiety (LAS score)	17.11 ± 8.81
Stress (BDS score)	13.79 ± 7.04
PTSD from COVID-19 pandemic	37.62 ± 15.78
PTSD from Beirut Blast	36.25 ± 15.73
PTSD from economic crisis	42.22 ± 17.97
Dissociative experiences scores	28.11 ± 19.29

### Factorial validity (confirmatory factor analysis of different models)

**Fig 1:**
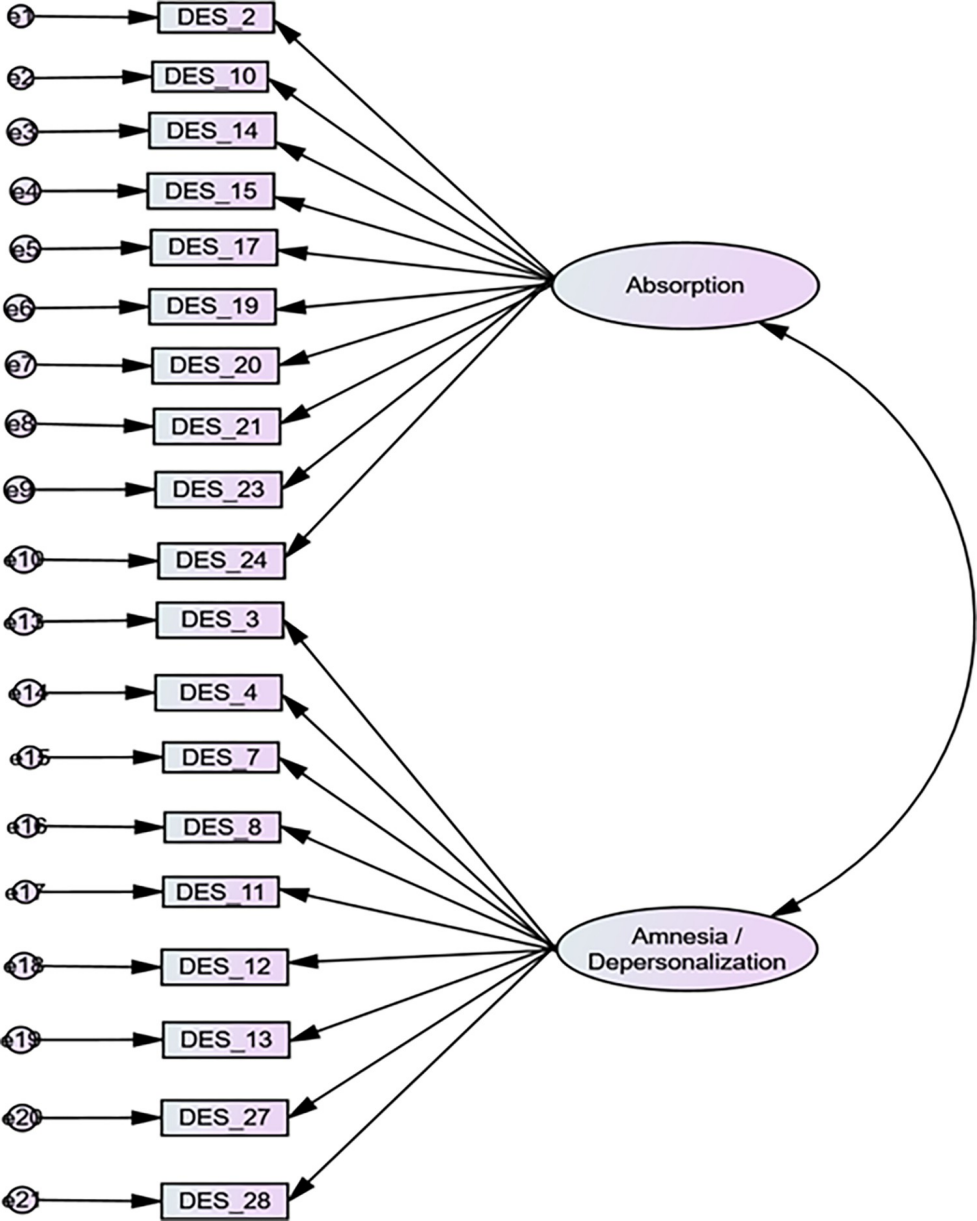
Standardized factor loadings of two-factor model of the Dissociative Experience Scale. *p<0.001.

The fit indices of the three tested CFA models are presented in Table 2. The two-factor model fitted best according to CFI, RMSEA and χ^2^/df values, but modestly according to TLI. The two factors were absorption and amnesia/depersonalization. The Cronbach’s alpha values for the absorption and amnesia/depersonalization were 0.892 and 0.895 respectively. The standardized factor loadings of the two-factor model of the DES are presented in Fig 1.

**Table 2:** Fit indices of the three tested confirmatory factor analyses models of the Dissociative Experience Scale (DES).

	χ^2^/df	TLI	CFI	RMSEA	90%CI
Model 1	1941.44/351 = 3.53	0.72	0.76	0.094	0.089–0.098
Model 2	557.89/152 = 3.67	0.87	0.90	0.072	0.065–0.078
Model 3	1381.41/350 = 3.95	0.82	0.84	0.075	0.071–0.080

*Note*: Model 1 = one factor [50, 51]; Model 2 = two factors [52]; Model 3 = Three factors [53].

### Divergent validity

Higher absorption and amnesia/depersonalization scores were positively associated with higher PTSD due to the Beirut blast (r = 0.481; p < .001 and r = 0.451; p<0.001).

### Bivariate analysis

Higher depression, anxiety, stress, PTSD from COVID-19 pandemic, from the Beirut blast, and from the economic crisis were significantly associated with higher absorption and amnesia/depersonalization, whereas a better financial wellbeing was significantly associated with less absorption and amnesia/depersonalization (Table 3). On another hand, higher mean dissociative experiences scores were found in those who have a personal history of anxiety, PTSD and depression compared to those who do not (Table 4).

**Table 3:** Correlation between the dissociative experiences score and other continuous variables.

	Absorption		Amnesia/depersonalization	
Variable	R	*p*	r	*p*
Age	-0.008	0.874	0.066	0.180
Household crowding index	-0.014	0.768	-0.015	0.761
Financial wellbeing scale	-0.230	**<0.001**	-0.136	**0.005**
Depression (PHQ-9 score)	0.466	**<0.001**	0.364	**<0.001**
Anxiety (LAS score)	0.504	**<0.001**	0.366	**<0.001**
Stress (BDS score)	0.505	**<0.001**	0.392	**<0.001**
PTSD from COVID-19 pandemic	0.489	**<0.001**	0.438	**<0.001**
PTSD from Beirut Blast	0.481	**<0.001**	0.451	**<0.001**
PTSD from economic crisis	0.489	**<0.001**	0.426	**<0.001**

Numbers in bold indicate significant p-values.

**Table 4:** Correlation between the dissociative experiences score and other categorical variables.

	Absorption			Amnesia/depersonalization		
Variable	Mean ± SD	*p*	Effect size	Mean ± SD	*p*	Effect size
**Gender**		0.043	0.219		0.779	0.030
Male	35.81 ± 22.28			17.02 ± 18.69		
Female	40.76 ± 22.87			16.47 ± 17.91		
**Personal history of anxiety disorders**		**<0.001**	0.597		**<0.001**	0.511
No	36.70 ± 22.40			14.74 ± 17.11		
Yes	49.78 ± 21.40			24.29 ± 20.10		
**Personal history of post-traumatic stress disorder**		**0.012**	0.530		**<0.001**	0.640
No	38.57 ± 22.74			15.84 ± 17.56		
Yes	50.19 ± 21.04			28.65 ± 22.20		
**Personal history of depression**		**<0.001**	0.498		**<0.001**	0.633
No	37.08 ± 22.13			14.27 ± 16.43		
Yes	48.40 ± 23.30			26.34 ± 21.37		

Numbers in bold indicate significant p values.

### Multivariable analysis

The results of a stepwise linear regression, taking the total dissociative experiences score as the dependent variable, showed that more PTSD from the Beirut blast (Beta = 0.32) and from the economic crisis (Beta = 0.15), more stress (Beta = 0.70), and having a personal history of PTSD (Beta = 6.37) were significantly associated with more dissociative experiences (Table 5, Model 1).

**Table 5:** Multivariable analyses.

**Model 1: Stepwise linear regression taking the total dissociative experiences scale score as the dependent variable.**				
**Variable**	**Unstandardized Beta (B)**	**Standardized Beta (β)**	***p***	**95% CI**
PTSD from Beirut Blast	0.32	0.26	<0.001	0.18–0.45
Stress	0.70	0.26	<0.001	0.41–0.99
PTSD from economic crisis	0.15	0.14	0.023	0.02–0.28
Personal history of PTSD	6.37	0.08	0.049	0.03–12.71
Variables entered in the model: PTSD from Beirut Blast, Stress (BDS score), PTSD from economic crisis, Personal history of PTSD, Personal history of anxiety disorder, Personal history of depression, Depression (PHQ9 score), Anxiety (LAS score), PTSD from COVID-19 pandemic, PTSD from Beirut Blast; PTSD = Post traumatic stress disorder. Nagelkerke R^2^ = 34.4%.				
**Model 2: Stepwise linear regression taking the absorption score as the dependent variable.**				
Stress	0.95	0.29	<0.001	0.61–1.28
PTSD from Beirut Blast	0.29	0.20	0.001	0.11–0.46
PTSD from COVID-19 pandemic	0.23	0.16	0.012	0.05–0.42
Variables entered in the model: PTSD from Beirut Blast, Stress (BDS score), PTSD from economic crisis, Personal history of PTSD, Personal history of anxiety disorder, Personal history of depression, Depression (PHQ9 score), Anxiety (LAS score), PTSD from COVID-19 pandemic, PTSD from Beirut Blast; PTSD = Post traumatic stress disorder. Nagelkerke R^2^ = 32.2%.				
**Model 3: Stepwise linear regression taking the amnesia/depersonalization score as the dependent variable.**				
PTSD from Beirut Blast	0.27	0.24	<0.001	0.13–0.42
Stress	0.36	0.14	0.013	0.08–0.64
Personal history of depression	6.03	0.13	0.003	2.05–10.00
PTSD from COVID-19 pandemic	0.16	0.14	0.035	0.01–0.32
Variables entered in the model: PTSD from Beirut Blast, Stress (BDS score), PTSD from economic crisis, Personal history of PTSD, Personal history of anxiety disorder, Personal history of depression, Depression (PHQ9 score), Anxiety (LAS score), PTSD from COVID-19 pandemic, PTSD from Beirut Blast; PTSD = Post traumatic stress disorder. Nagelkerke R^2^ = 25.8%.				

Higher stress (Beta = 0.95) and more PTSD from the Beirut blast (Beta = 0.29) and from the economic crisis (Beta = 0.23) were significantly associated with more absorption (Table 5, Model 2).

A personal history of depression (Beta = 6.03), higher stress (Beta = 0.36) and more PTSD from the Beirut blast (Beta = 0.27) and from the COVID-19 pandemic (Beta = 0.16) were significantly associated with more amnesia/depersonalization (Table 5, Model 3).

## Discussion

### Dissociative experiences and PTSD

Dissociation has been linked to a wide range of mental illnesses [6]. Several sources of evidence point to a strong link between dissociation and psychological trauma, particularly cumulative and/or early childhood trauma [54]. From a period of relative obscurity during much of the twentieth century to a recent resurgence of interest in the role of dissociation in understanding humans’ responses to traumatic events, research has progressed far enough to address not just the relationship between trauma and dissociative tendencies in general [55], but also to propose the hypothesis of a dissociative subtype of post-traumatic stress disorder (D-PTSD) [56]. The existence of cumulative/repetitive early life trauma is one of the criteria that is present in D-PTSD and subsequently distinguishes it from non-D-PTSD [57]. This previous information is consistent with our findings, which demonstrate an association between a personal history of PTSD and dissociative experiences in a relatively young population, especially given that the national PTSD rate in Lebanon is approaching the 25% mark [58]. Dissociative episodes, on the other hand, may raise the likelihood of repeated traumatization by flattening the surge of defensive reaction mechanisms to various stressors, thus increasing the chance of the development of a full blown post-traumatic stress disorder [59, 60]. In our study, however, a single traumatic event, such as post-traumatic manifestations from the Beirut blast and/or the current Lebanese economic crisis, was also found to be strongly linked to greater dissociative experiences.

From a socio-economic point of view, the elevated level of stress around millennials’ finances has been demonstrated to cause pathological repercussions on their thoughts, feelings, and behaviors, which are most typically connected with post-traumatic stress disorder (PTSD) [61]. The emergence of peri-traumatic dissociation could be found in the setting of lower socio-economic status, as revealed in a study among Latinos about racial/ethnic conditional risks for PTSD [62]. While low socio-economic status could be seen as a conditional risk by itself for dissociative experiences, a traumatic change in the socio-economic status like the consequence of the rapid Lebanese economic crisis would be considered as an additional explanation for dissociation [62]. In fact, a change in the socio-economic crisis may be perceived as a possible identity change, where dissociative experiences would be exhibited as a possible adaptation mechanism, as explained in a parallel but previous study about dissociative symptoms in a Hungarian population sample [63].

Furthermore, a catastrophic yet devastating trauma as the Beirut blast, which may be perceived as a thunderclap disfiguration of a geography, a nation, and a population, has thrown the Lebanese people in a possible existential *split identity* [64–66]. In the face of such collective trauma and subsequent decompensation, the Lebanese population response could range from a routine acceptance of the “status quo” to a more complex adaptation such as dissociative experiences. Indeed, dissociation is implemented as a defense mechanism preventing the processing of raw data, in a way that memories are fragmented before they are stored [67], and hence trauma-related memories would appear different from non-traumatic ones as of intrusiveness, amnesias, and vividness [68]. The latter form of adaptation could be understood by a comparable response of victims of other genocides that recently happened in countries such as Nigeria, Rwanda, former Yugoslavia, Cambodia, and Sudan [68, 69].

Our results also found a correlation between dissociative absorption and PTSD. This might appear surprising because this symptom has been widely accepted as normative dissociation because absorption is linked to creative, hypnotic, and other non-pathological processes, whereas other dissociative events are more intimately linked to psychopathology [70]. It has been demonstrated that there is a modest positive relationship between absorption and Post Traumatic Stress in less traumatized populations, but not in highly traumatized groups [70]. Furthermore, some findings indicated that greater absorption could help predict the diagnosis of PTSD: those results support the theory that absorption may be a risk factor for the development of dissociation and posttraumatic stress symptoms following traumatic exposure [71]. On a physiological level, a laboratory study by Giesbrecht et al. (2008) indicated that people who have a high absorption score are more prone to having heightened rates of peritraumatic dissociation following painful stimulation caused by a cold pressor test [72].

Dissociative amnesia was also associated with PTSD, which is plausible given that amnesia is a documented comorbidity of PTSD knowing that some patients with an initial clinical presentation of dissociative amnesia are often re-diagnosed with PTSD [73, 74]. Major stress and trauma in which the patient was unable to cope are suspected to be the etiology [74, 75]. Some papers also linked the cause of dissociative amnesia to a history of a stressful youth, as well as a major traumatic incident in the past and an additional proximal distressing event [76, 77].

As for depersonalization and PTSD, a positive relationship was identified between the two variables, which is also supported by previous studies where a robust correlation between Depersonalization/Derealization and Post Traumatic Stress was established [70]. In addition, a paper studying the relationship between PTSD and Dissociation found that adolescents who have been subjected to mistreatment tend to have a co-occurrence of PTSD and depersonalization/derealization at higher rates than adult [78]. The COVID-19 pandemic had a significant psychological impact on university students around the world [79] and in Lebanon, according to our study and previous research [80]. Moreover, our results presented high rates of PTSD from the Beirut Blast. That’s why the coexistence of PTSD and Derealization/Depersonalization may not be surprising, given that the more severe the post-traumatic symptoms are, the more pronounced is the presentation of Derealization/Depersonalization [81]. Contrastingly, it’s also worth noting that what the youth perceive as depersonalization/derealization may be emotional numbing rather than dissociation [82].

### Dissociative experiences and stress

Our study found an association between dissociative experiences and high levels of stress. Which is consistent with previous research. In fact, dissociative experiences have been highly correlated to acute and/or chronic stress [6, 83]. Dissociative symptoms are very frequently observed in healthy people exposed to a lot of stress. Dissociative states can be described as an acute and/or ephemeral reaction to stressful life events as well as interpersonal issues [9]. A review about the many faces of dissociation [83] adding more depth to the previous statement, also revealed that dissociation is the eventual response of a human to a certain chronic stress. It has been reported that university students suffer from greater levels of stress as compared to the general population [84]. Furthermore, the COVID-19 pandemic was a significant aggravator of distress among college students [85], which may in part explain the high degree of stress in our study group and, as a result, its link to dissociative symptoms.

Our results also revealed that amnesia, a sub-factor of the Dissociative Experiences Scale and a major display of dissociation, was strongly linked with stress. According to the ICD-10, stress and trauma play significant roles in the development of dissociative amnesia [86]. The exact mechanism by which persistent stress causes dissociative amnesia is not yet fully understood, but some studies showed that persistent glucocorticoid levels have been associated to poor memory performance [87]. According to some epigenetic models, the consequence of gene-environment interactions prior to the onset of amnesia can increase the risk of developing dissociative amnesia following trauma or psychological stress [88–90].

Our findings demonstrated that, like dissociative amnesia, absorption and derealization/depersonalization were also substantially associated to stress. There is a scarcity of data surrounding absorption and its association to stress, but some studies revealed that absorption was associated to stress and, more specifically to comorbid obsessive-compulsive symptoms [91].

As for depersonalization, our results showed that it has a substantial association with stress, as seen in previous studies [92, 93]. Stress has been recognized as a primary precipitant of depersonalization/derealization, but once this latter state reaches chronicity, stress isn’t even detectable in some cases [94, 95]. The links between absorption and distress are hazy, and additional research suggest that absorption has a negative relationship with stress when contrasted to depersonalization/derealization [92]. On a comparative note, depersonalization/derealization, considered as a malignant expression of dissociation, was linked to higher cortisol levels, but the benign forms of dissociation, such as absorption, was linked to lower cortisol levels [92]. Indeed, it was also demonstrated that there is a significant link between absorption and subjective stress, but those results, as mentioned above, failed to translate into physiological stress (cortisol levels) knowing the association came back negative [92, 96]. It’s suggested that the elevated levels of stress hormones in depersonalization/derealization is possibly due to decreased negative feedback on the hypothalamic-hypopituitary axis but, the lower cortisol levels seen in absorption can be explained by some findings suggesting that people who dissociate a lot during moments of stress have less peripheral stress-related physiology during the acute stressful period [92]. Another issue concerns the clinical nature of dissociative absorption: the two other factors of dissociation are deeply rooted with distinct clinical disorders such as depersonalization-derealization disorder and dissociative amnesia disorder [1], but in the other hand there is no clinical disorder that matches with absorption. This has led some to consider that dissociation could possibly be divided to two branches: the clinical-based (mostly related to trauma) and the non-clinical or non-pathological type of dissociation (described as a normal process, mild-type of dissociation, linked mainly to character trait) [97]. Ultimately, we used the two-factor model in this study because it encompasses two distinct types of dissociative experiences that differ qualitatively and in severity. In conclusion, we believe that more research into Dissociative Absorption and its correlates is needed to properly categorize it and determine its clinical significance.

### Dissociative experiences and a personal history of depression

Our findings also point to a relationship between dissociative amnesia and depersonalization with a personal history of depression. A paper analyzing the link between child sexual abuse and dissociative symptoms also back up our data, indicating that there was a strong link between dissociative amnesia and depression in their sample [7]. Amnesia was also shown to interact with the intensity of a pre-existing depression by exacerbating the latter [8], which also supports our results.

Additionally, depersonalization is frequently associated with depressive states, as they are both common comorbidities [98, 99]. A sample from the prospective Gutenberg Health Study assessed the psychological correlates of depersonalization/derealization: The symptoms of derealization were strongly associated with the prevalence of depression at the 2.5-year follow-up. This longitudinal research found that derealization symptoms are linked to the severity of mental distress and are related with a higher likelihood of poor outcome [100].

### Clinical implications

Significant rates of dissociative experiences (Absorption, Amnesia/Depersonalization) were found among Lebanese university students in the current study, along with a striking co-occurrence of traumatic/stressful patterns, whether on an individual (PTSD history) or collective level (PTSD from Beirut blast, COVID-19 pandemic, and/or economic crisis), or whether correlated to an acute single event, certain chronic stressors (BDS-10) or a personal history of depression. While Lebanese practitioners are regarded as front-line responders to victims of the abovementioned traumatic incidents for non-psychological problems, our article shed light on the possibility of underlying and/or co-occurring PTSD and PTSD-D experiences. Thus, health managers and physicians must categorize specific risk factors and develop evidence-based interventions to predict the risk of the settlement of dissociative experiences and/or PTSD, as well as provide preventive and diagnostic initiatives in order to deliver subsequent high-quality care for the affected population.

### Limitations

This study, like every research, has several limitations. First, the cross-sectional design of this research makes it difficult to ascertain causality, and it also makes it hard to rule out the potential that our observations were influenced by variables not investigated in our study. Second, the data was collected entirely through self-reported measures rather than physician diagnosis, which means that the accuracy of individual reports cannot be guaranteed, and interpretations should be made with caution, even though the measures used in this study have been widely employed in research and have consistently demonstrated adequate psychometric properties. Further assessments by mental health professionals are needed to confirm the results obtained. Third, snowball sampling and respondent-driven sampling techniques were used because of the COVID-19-related social distancing, which predisposes us to selection biases. Fourth, this study type is subject to an information bias due to inaccurate information from participants. Fifth, in our sample females were more represented numerically than males: Future studies with an equal number of males and females will be needed to assess the relationship between gender and the studied variables. Sixth, there was scarcity of data in literature to support our findings, knowing that some our results derive from theory with no concise explanation. Finally, our sample was limited to university students only, so participants were primary young, highly educated and not representative of the whole Lebanese population.

Because of the aforementioned constraints, the findings of this study are not generalizable to the entire population. Clinical sample studies are desperately needed to expand our findings and give more definitive public health implications.

## Conclusion

University students in Lebanon were exposed to very severe stressors that is why it was crucial to investigate the traumatic repercussions of the COVID-19 pandemic, the Beirut Blast and the Economic Crisis and the subsequent development of Dissociative Experiences and their correlates. In our paper, dissociative experiences and their sub-manifestations (amnesia/depersonalization and absorption) were found at significantly high rates among Lebanese university students, with remarkable co-occurrence of a traumatic/stressful pattern, whether on an individual (history of PTSD) or a collective level (PTSD from Beirut blast, COVID-19 pandemic and/or economic crisis), or whether correlated to an acute single event, to certain chronic stressors or a personal history of depression. However, these results remain limited to a restrained sample of Lebanese university students. Nonetheless, such findings must raise the attention to serious mental and psychosocial alteration in the Lebanese national identity. When dealing with dissociative experiences, several aspects should be taken into consideration. The lack of a consistent set of symptoms and overlap with other psychiatric diseases and diagnosis can be difficult to determine. That is why patients are frequently lately diagnosed and have already experienced the effects of the condition, such as self-harm, disability, and loss of employment. Those repercussions are detrimental on a population of university students, which are considered the future of society. Therefore, further studies should be done on the general population regarding the relationship between depressive disorders and dissociative experiences, as well as the impact of stress on the onset of dissociative symptoms. Moreover, more research is warranted about the dissociation subscales, particularly dissociative amnesia, depersonalization/derealization, and absorption to better categorize them and determine their clinical significance. Trauma and dissociation have received a lot of attention in research, but other plausible origins of the disorder have been overlooked or at least were given less importance. Whether the Lebanese population, collectively or individually is enduring dissociative experiences or not there is a need of future investigations and research, through future longitudinal and prospective studies. Consequently, our findings must raise awareness to the substantial mental and emotional challenges that Lebanon’s youth are experiencing. Therefore, screenings, preventative programs and diagnostic measures are encouraged for implementation by decision makers, researchers, and stakeholders on a nationwide level.

## References

[1] American Psychiatric Association A. Diagnostic and statistical manual of mental disorders- 5th Edition: American Psychiatric Association Washington, DC; 2013.

[2] Soffer-Dudek N. Dissociative absorption, mind-wandering, and attention-deficit symptoms: Associations with obsessive-compulsive symptoms. Br J Clin Psychol. 2019;58(1):51–69. doi: 10.1111/bjc.12186 . 29873088

[3] Soffer-Dudek N, Lassri D, Soffer-Dudek N, Shahar G. Dissociative absorption: An empirically unique, clinically relevant, dissociative factor. Conscious Cogn. 2015;36:338–51. doi: 10.1016/j.concog.2015.07.013 . 26241024

[4] Kate MA, Hopwood T, Jamieson G. The prevalence of Dissociative Disorders and dissociative experiences in college populations: a meta-analysis of 98 studies. J Trauma Dissociation. 2020;21(1):16–61. doi: 10.1080/15299732.2019.1647915 . 31461395

[5] Calati R, Bensassi I, Courtet P. The link between dissociation and both suicide attempts and non-suicidal self-injury: Meta-analyses. Psychiatry Res. 2017;251:103–14. doi: 10.1016/j.psychres.2017.01.035 . 28196773

[6] Lyssenko L, Schmahl C, Bockhacker L, Vonderlin R, Bohus M, Kleindienst N. Dissociation in Psychiatric Disorders: A Meta-Analysis of Studies Using the Dissociative Experiences Scale. Am J Psychiatry. 2018;175(1):37–46. doi: 10.1176/appi.ajp.2017.17010025 . 28946763

[7] Wolf MR, Nochajski TH. Child sexual abuse survivors with dissociative amnesia: what’s the difference? J Child Sex Abus. 2013;22(4):462–80. doi: 10.1080/10538712.2013.781094 . 23682770

[8] Kilic O, Sar V, Taycan O, Aksoy-Poyraz C, Erol TC, Tecer O, et al. Dissociative depression among women with fibromyalgia or rheumatoid arthritis. J Trauma Dissociation. 2014;15(3):285–302. doi: 10.1080/15299732.2013.844218 . 24228798

[9] Morgan CA 3rd, Hazlett G, Wang S, Richardson EG Jr., Schnurr P, Southwick SM. Symptoms of dissociation in humans experiencing acute, uncontrollable stress: a prospective investigation. Am J Psychiatry. 2001;158(8):1239–47. doi: 10.1176/appi.ajp.158.8.1239 . 11481157

[10] Evren C, Sar V, Dalbudak E. Temperament, character, and dissociation among detoxified male inpatients with alcohol dependency. J Clin Psychol. 2008;64(6):717–27. doi: 10.1002/jclp.20485 . 18384114

[11] Sareen J. Posttraumatic stress disorder in adults: impact, comorbidity, risk factors, and treatment. Can J Psychiatry. 2014;59(9):460–7. doi: 10.1177/070674371405900902 ; PubMed Central PMCID: PMC4168808. 25565692 PMC4168808

[12] Liu CH, Zhang E, Wong GTF, Hyun S, Hahm HC. Factors associated with depression, anxiety, and PTSD symptomatology during the COVID-19 pandemic: Clinical implications for U.S. young adult mental health. Psychiatry Res. 2020;290:113172. doi: 10.1016/j.psychres.2020.113172 ; PubMed Central PMCID: PMC7263263. 32512357 PMC7263263

[13] Shaar KH. Post-traumatic stress disorder in adolescents in Lebanon as wars gained in ferocity: a systematic review. J Public Health Res. 2013;2(2):e17. doi: 10.4081/jphr.2013.e17 ; PubMed Central PMCID: PMC4147728. 25170488 PMC4147728

[14] Muris P, Merckelbach H, Peeters E. The links between the Adolescent Dissociative Experiences Scale (A-DES), fantasy proneness, and anxiety symptoms. J Nerv Ment Dis. 2003;191(1):18–24. doi: 10.1097/00005053-200301000-00004 . 12544595

[15] El Hajj M. Prevalence and associated factors of post-traumatic stress disorder in Lebanon: A literature review. Asian J Psychiatr. 2021;63:102800. doi: 10.1016/j.ajp.2021.102800 . 34340165

[16] Jaju S, Al-Adawi S, Al-Kharusi H, Morsi M, Al-Riyami A. Prevalence and age-of-onset distributions of DSM IV mental disorders and their severity among school going Omani adolescents and youths: WMH-CIDI findings. Child and Adolescent Psychiatry and Mental Health. 2009;3(1):1–11. 19781098 10.1186/1753-2000-3-29 PMC2761855

[17] El Zouki CJ, Chahine A, Mhanna M, Obeid S, Hallit S. Rate and correlates of post-traumatic stress disorder (PTSD) following the Beirut blast and the economic crisis among Lebanese University students: a cross-sectional study. BMC Psychiatry. 2022;22(1):532. doi: 10.1186/s12888-022-04180-y ; PubMed Central PMCID: PMC9356397. 35931970 PMC9356397

[18] Consultancy-me.com. Lebanon’s unemployment rate surges past 30% amid meltdown. Available from: https://www.consultancy-me.com/news/2900/lebanons-unemployment-rate-surges-past-30-amid-meltdown.

[19] El Othman R, Touma E, El Othman R, Haddad C, Hallit R, Obeid S, et al. COVID-19 pandemic and mental health in Lebanon: a cross-sectional study. Int J Psychiatry Clin Pract. 2021;25(2):152–63. doi: 10.1080/13651501.2021.1879159 . 33587678

[20] Ran L, Wang W, Ai M, Kong Y, Chen J, Kuang L. Psychological resilience, depression, anxiety, and somatization symptoms in response to COVID-19: A study of the general population in China at the peak of its epidemic. Soc Sci Med. 2020;262:113261. doi: 10.1016/j.socscimed.2020.113261 ; PubMed Central PMCID: PMC7388777. 32758794 PMC7388777

[21] Ko NY, Lu WH, Chen YL, Li DJ, Wang PW, Hsu ST, et al. COVID-19-related information sources and psychological well-being: An online survey study in Taiwan. Brain Behav Immun. 2020;87:153–4. doi: 10.1016/j.bbi.2020.05.019 ; PubMed Central PMCID: PMC7204755 competing financial interests or personal relationships that could have appeared to influence the work reported in this paper. 32389702 PMC7204755

[22] Hashim HT, Uakkas S, Reda A, Ramadhan MA, Al Mostafa MY. Beirut Explosion Effects on COVID-19 Situation in Lebanon. Disaster Med Public Health Prep. 2021:1–2. doi: 10.1017/dmp.2021.56 ; PubMed Central PMCID: PMC8111190. 33588973 PMC8111190

[23] Hussein B. BSI economics. Large Devaluation and Inequality: The Case of Lebanon (Note). http://www.bsi-economics.org/1130-large-develuation-inquality-case-of-lebanon. 2020.

[24] Lahoud N, Zakhour M, Haddad C, Salameh P, Akel M, Fares K, et al. Burnout and Its Relationships With Alexithymia, Stress, Self-Esteem, Depression, Alcohol Use Disorders, and Emotional Intelligence: Results From a Lebanese Cross-Sectional Study. J Nerv Ment Dis. 2019;207(8):642–50. doi: 10.1097/NMD.0000000000001017 . 31356406

[25] Obeid S, Akel M, Haddad C, Fares K, Sacre H, Salameh P, et al. Factors associated with alexithymia among the Lebanese population: results of a cross-sectional study. BMC Psychol. 2019;7(1):80. doi: 10.1186/s40359-019-0353-5 ; PubMed Central PMCID: PMC6907355. 31829280 PMC6907355

[26] Obeid S, Akel M, Haddad C, Fares K, Sacre H, Salameh P, et al. Factors associated with alcohol use disorder: the role of depression, anxiety, stress, alexithymia and work fatigue- a population study in Lebanon. BMC Public Health. 2020;20(1):245. doi: 10.1186/s12889-020-8345-1 ; PubMed Central PMCID: PMC7029557. 32070314 PMC7029557

[27] Obeid S, Lahoud N, Haddad C, Sacre H, Akel M, Fares K, et al. Factors associated with depression among the Lebanese population: Results of a cross-sectional study. Perspect Psychiatr Care. 2020;56(4):956–67. doi: 10.1111/ppc.12518 . 32314394

[28] Obeid S, Lahoud N, Haddad C, Sacre H, Fares K, Akel M, et al. Factors associated with anxiety among the Lebanese population: the role of alexithymia, self-esteem, alcohol use disorders, emotional intelligence and stress and burnout. Int J Psychiatry Clin Pract. 2020;24(2):151–62. doi: 10.1080/13651501.2020.1723641 . 32031427

[29] Zakhour M, Haddad C, Sacre H, Fares K, Akel M, Obeid S, et al. Suicidal ideation among Lebanese adults: scale validation and correlates. BMC Psychiatry. 2021;21(1):100. doi: 10.1186/s12888-021-03111-7 ; PubMed Central PMCID: PMC7888108. 33593321 PMC7888108

[30] Chahine M, Salameh P, Haddad C, Sacre H, Soufia M, Akel M, et al. Suicidal ideation among Lebanese adolescents: scale validation, prevalence and correlates. BMC Psychiatry. 2020;20(1):304. doi: 10.1186/s12888-020-02726-6 ; PubMed Central PMCID: PMC7296775. 32539735 PMC7296775

[31] Merhy G, Azzi V, Salameh P, Obeid S, Hallit S. Anxiety among Lebanese adolescents: scale validation and correlates. BMC Pediatr. 2021;21(1):288. doi: 10.1186/s12887-021-02763-4 ; PubMed Central PMCID: PMC8218523. 34158020 PMC8218523

[32] Sfeir E, Geara C, Hallit S, Obeid S. Alexithymia, aggressive behavior and depression among Lebanese adolescents: A cross-sectional study. Child Adolesc Psychiatry Ment Health. 2020;14:32. doi: 10.1186/s13034-020-00338-2 ; PubMed Central PMCID: PMC7487493. 32939221 PMC7487493

[33] Sfeir E, Haddad C, Akel M, Hallit S, Obeid S. Sleep disorders in a sample of Lebanese children: the role of parental mental health and child nutrition and activity. BMC Pediatr. 2021;21(1):324. doi: 10.1186/s12887-021-02795-w ; PubMed Central PMCID: PMC8298696. 34301219 PMC8298696

[34] Wang X, Hegde S, Son C, Keller B, Smith A, Sasangohar F. Investigating Mental Health of US College Students During the COVID-19 Pandemic: Cross-Sectional Survey Study. J Med Internet Res. 2020;22(9):e22817. doi: 10.2196/22817 ; PubMed Central PMCID: PMC7505693. 32897868 PMC7505693

[35] Pedrelli P, Nyer M, Yeung A, Zulauf C, Wilens T. College Students: Mental Health Problems and Treatment Considerations. Acad Psychiatry. 2015;39(5):503–11. doi: 10.1007/s40596-014-0205-9 ; PubMed Central PMCID: PMC4527955. 25142250 PMC4527955

[36] Maraldi EO. Temporal Stability of Dissociative Symptoms in the General Population. J Nerv Ment Dis. 2022;210(1):68–70. doi: 10.1097/NMD.0000000000001411 . 34982752

[37] Muthén LK, Muthén BO. How to use a Monte Carlo study to decide on sample size and determine power. Structural equation modeling. 2002;9(4):599–620.

[38] Carlson EB, Putnam FW. An update on the dissociative experiences scale. Dissociation: progress in the dissociative disorders. 1993.

[39] Wilkins KC, Lang AJ, Norman SB. Synthesis of the psychometric properties of the PTSD checklist (PCL) military, civilian, and specific versions. Depression and anxiety. 2011;28(7):596–606. doi: 10.1002/da.20837 21681864 PMC3128669

[40] Prawitz A, Garman ET, Sorhaindo B, O’Neill B, Kim J, Drentea P. InCharge financial distress/financial well-being scale: Development, administration, and score interpretation. Journal of Financial Counseling and Planning. 2006;17(1).

[41] Malaeb D, Farchakh Y, Haddad C, Sacre H, Obeid S, Hallit S, et al. Validation of the Beirut Distress Scale (BDS-10), a short version of BDS-22, to assess psychological distress among the Lebanese population. Perspect Psychiatr Care. 2021. doi: 10.1111/ppc.12787 . 33821486

[42] Hallit S, Obeid S, Haddad C, Hallit R, Akel M, Haddad G, et al. Construction of the Lebanese Anxiety Scale (LAS-10): a new scale to assess anxiety in adult patients. Int J Psychiatry Clin Pract. 2020;24(3):270–7. doi: 10.1080/13651501.2020.1744662 . 32228282

[43] Kroenke K, Spitzer RL, Williams JB. The PHQ-9: validity of a brief depression severity measure. J Gen Intern Med. 2001;16(9):606–13. doi: 10.1046/j.1525-1497.2001.016009606.x ; PubMed Central PMCID: PMC1495268. 11556941 PMC1495268

[44] Sawaya H, Atoui M, Hamadeh A, Zeinoun P, Nahas Z. Adaptation and initial validation of the Patient Health Questionnaire—9 (PHQ-9) and the Generalized Anxiety Disorder—7 Questionnaire (GAD-7) in an Arabic speaking Lebanese psychiatric outpatient sample. Psychiatry Res. 2016;239:245–52. doi: 10.1016/j.psychres.2016.03.030 . 27031595

[45] Byrne BM. Structural equation modeling with Mplus: Basic concepts, applications, and programming: routledge; 2013.

[46] Marsh HW, Hau K-T, Wen Z. In search of golden rules: Comment on hypothesis-testing approaches to setting cutoff values for fit indexes and dangers in overgeneralizing Hu and Bentler’s (1999) findings. Structural equation modeling. 2004;11(3):320–41.

[47] George D. SPSS for windows step by step: A simple study guide and reference, 17.0 update, 10/e: Pearson Education India; 2011.

[48] Mishra P, Pandey CM, Singh U, Gupta A, Sahu C, Keshri A. Descriptive statistics and normality tests for statistical data. Ann Card Anaesth. 2019;22(1):67–72. doi: 10.4103/aca.ACA_157_18 ; PubMed Central PMCID: PMC6350423. 30648682 PMC6350423

[49] Vandekerckhove J, Matzke D, Wagenmakers E-J. Model comparison and the principle of parsimony: eScholarship, University of California; 2014.

[50] Fischer DG, Elnitsky S. A factor analytic study of two scales measuring dissociation. American Journal of Clinical Hypnosis. 1990;32(3):201–7. doi: 10.1080/00029157.1990.10402825 2296922

[51] Holtgraves T, Stockdale G. The assessment of dissociative experiences in a non-clinical population: Reliability, validity, and factor structure of the Dissociative Experiences Scale. Personality and Individual differences. 1997;22(5):699–706.

[52] Olsen SA, Clapp JD, Parra GR, Beck JG. Factor structure of the Dissociative Experiences Scale: An examination across sexual assault status. Journal of psychopathology and behavioral assessment. 2013;35(3):394–403.

[53] Ross CA, Ellason JW, Anderson G. A factor analysis of the Dissociative Experiences Scale (DES) in dissociative identity disorder. Dissociation: progress in the dissociative disorders. 1995.

[54] Loewenstein RJ. Dissociation debates: Everything you know is wrong. Dialogues in clinical neuroscience. 2022. 10.31887/DCNS.2018.20.3/rloewenstein PMC6296396 30581293

[55] Wilson JP, Tang CCS-K. Cross-cultural assessment of psychological trauma and PTSD: Springer Science & Business Media; 2007.

[56] Cramer AOJ, Leertouwer I, Lanius R, Frewen P. A Network Approach to Studying the Associations Between Posttraumatic Stress Disorder Symptoms and Dissociative Experiences. J Trauma Stress. 2020;33(1):19–28. doi: 10.1002/jts.22488 ; PubMed Central PMCID: PMC7154636. 32086973 PMC7154636

[57] van Huijstee J, Vermetten E. The Dissociative Subtype of Post-traumatic Stress Disorder: Research Update on Clinical and Neurobiological Features. Curr Top Behav Neurosci. 2018;38:229–48. doi: 10.1007/7854_2017_33 . 29063485

[58] Farhood LF, Fares S, Sabbagh R, Hamady C. PTSD and depression construct: prevalence and predictors of co-occurrence in a South Lebanese civilian sample. Eur J Psychotraumatol. 2016;7:31509. doi: 10.3402/ejpt.v7.31509 ; PubMed Central PMCID: PMC4944596. 27414815 PMC4944596

[59] Bockers E, Roepke S, Michael L, Renneberg B, Knaevelsrud C. Risk recognition, attachment anxiety, self-efficacy, and state dissociation predict revictimization. PLoS One. 2014;9(9):e108206. doi: 10.1371/journal.pone.0108206 ; PubMed Central PMCID: PMC4169587. 25238153 PMC4169587

[60] Zamir O, Szepsenwol O, Englund MM, Simpson JA. The role of dissociation in revictimization across the lifespan: A 32-year prospective study. Child Abuse Negl. 2018;79:144–53. doi: 10.1016/j.chiabu.2018.02.001 . 29454258

[61] Businesswire. New Payoff Study Finds Nearly 1 in 4 Americans and 1 in 3 Millennials Suffer From PTSD-Like Symptoms Caused by Financially Induced Stress. Available from: https://www.businesswire.com/news/home/20160420005504/en/New-Payoff-Study-Finds-Nearly-1-in-4-Americans-and-1-in-3-Millennials-Suffer-From-PTSD-Like-Symptoms-Caused-by-Financially-Induced-Stress 2016.

[62] Alcantara C, Casement MD, Lewis-Fernandez R. Conditional risk for PTSD among Latinos: a systematic review of racial/ethnic differences and sociocultural explanations. Clin Psychol Rev. 2013;33(1):107–19. doi: 10.1016/j.cpr.2012.10.005 ; PubMed Central PMCID: PMC3535473. 23159328 PMC3535473

[63] Vanderlinden J, Varga K, Peuskens J, Pieters G. Dissociation: Vol. 8, No. 4, p. 205–208: Dissociative symptoms in a population sample of Hungary. 1995.

[64] Rakesh G, Morey RA, Zannas AS, Malik Z, Marx CE, Clausen AN, et al. Resilience as a translational endpoint in the treatment of PTSD. Mol Psychiatry. 2019;24(9):1268–83. doi: 10.1038/s41380-019-0383-7 ; PubMed Central PMCID: PMC6713904. 30867558 PMC6713904

[65] El Hayek S, Bizri M. Beirut blast and mental health in Lebanon: Finding ways out. Asian J Psychiatr. 2020;54:102458. doi: 10.1016/j.ajp.2020.102458 . 33271737

[66] Duckers ML, Brewin CR. A Paradox in Individual Versus National Mental Health Vulnerability: Are Higher Resource Levels Associated With Higher Disorder Prevalence? J Trauma Stress. 2016;29(6):572–6. doi: 10.1002/jts.22144 . 27859656

[67] Kennedy F, Clarke S, Stopa L, Bell L, Rouse H, Ainsworth C, et al. Towards a cognitive model and measure of dissociation. J Behav Ther Exp Psychiatry. 2004;35(1):25–48. doi: 10.1016/j.jbtep.2004.01.002 . 15157816

[68] Fridman A, Bakermans-Kranenburg MJ, Sagi-Schwartz A, Van IMH. Coping in old age with extreme childhood trauma: aging Holocaust survivors and their offspring facing new challenges. Aging Ment Health. 2011;15(2):232–42. doi: 10.1080/13607863.2010.505232 . 20924822

[69] Sandole DH, Auerbach CF. Dissociation and identity transformation in female survivors of the genocide against the Tutsi in Rwanda: a qualitative research study. J Trauma Dissociation. 2013;14(2):127–37. doi: 10.1080/15299732.2013.724345 . 23406218

[70] Cardena E, Gusic S, Cervin M. A Network Analysis to Identify Associations between PTSD and Dissociation among Teenagers. J Trauma Dissociation. 2021:1–19. doi: 10.1080/15299732.2021.1989122 . 34678139

[71] Simeon D, Giesbrecht T, Knutelska M, Smith RJ, Smith LM. Alexithymia, absorption, and cognitive failures in depersonalization disorder: a comparison to posttraumatic stress disorder and healthy volunteers. J Nerv Ment Dis. 2009;197(7):492–8. doi: 10.1097/NMD.0b013e3181aaef6b . 19597356

[72] Giesbrecht T, Smeets T, Merckelbach H. Dissociative experiences on ice—peritraumatic and trait dissociation during the cold pressor test. Psychiatry Res. 2008;157(1–3):115–21. doi: 10.1016/j.psychres.2006.12.012 . 17869347

[73] Clouden TA. Dissociative Amnesia and Dissociative Fugue in a 20-Year-Old Woman With Schizoaffective Disorder and Post-Traumatic Stress Disorder. Cureus. 2020;12(5):e8289. doi: 10.7759/cureus.8289 ; PubMed Central PMCID: PMC7255065. 32483516 PMC7255065

[74] Staniloiu A, Markowitsch HJ. Dissociative amnesia. The Lancet Psychiatry. 2014;1(3):226–41. doi: 10.1016/S2215-0366(14)70279-2 26360734

[75] Dalenberg CJ, Brand BL, Gleaves DH, Dorahy MJ, Loewenstein RJ, Cardena E, et al. Evaluation of the evidence for the trauma and fantasy models of dissociation. Psychol Bull. 2012;138(3):550–88. doi: 10.1037/a0027447 . 22409505

[76] MacDonald K, MacDonald T. Peas, please: a case report and neuroscientific review of dissociative amnesia and fugue. J Trauma Dissociation. 2009;10(4):420–35. doi: 10.1080/15299730903143618 . 19821177

[77] Markowitsch HJ. Psychogenic amnesia. Neuroimage. 2003;20 Suppl 1:S132–8. doi: 10.1016/j.neuroimage.2003.09.010 . 14597306

[78] Choi KR, Ford JD, Briggs EC, Munro-Kramer ML, Graham-Bermann SA, Seng JS. Relationships Between Maltreatment, Posttraumatic Symptomatology, and the Dissociative Subtype of PTSD Among Adolescents. J Trauma Dissociation. 2019;20(2):212–27. doi: 10.1080/15299732.2019.1572043 ; PubMed Central PMCID: PMC6407637. 30714854 PMC6407637

[79] Li X, Fu P, Fan C, Zhu M, Li M. COVID-19 Stress and Mental Health of Students in Locked-Down Colleges. Int J Environ Res Public Health. 2021;18(2). doi: 10.3390/ijerph18020771 ; PubMed Central PMCID: PMC7831318. 33477595 PMC7831318

[80] Kassir G, El Hayek S, Zalzale H, Orsolini L, Bizri M. Psychological distress experienced by self-quarantined undergraduate university students in Lebanon during the COVID-19 outbreak. Int J Psychiatry Clin Pract. 2021;25(2):172–9. doi: 10.1080/13651501.2021.1900872 . 33775208

[81] Hansen M, Ross J, Armour C. Evidence of the dissociative PTSD subtype: A systematic literature review of latent class and profile analytic studies of PTSD. J Affect Disord. 2017;213:59–69. doi: 10.1016/j.jad.2017.02.004 . 28192736

[82] Merritt RD, You S. Is there really a dissociative taxon on the dissociative experiences scale? J Pers Assess. 2008;90(2):201–3. doi: 10.1080/00223890701845492 . 18444115

[83] Sar V. The many faces of dissociation: opportunities for innovative research in psychiatry. Clin Psychopharmacol Neurosci. 2014;12(3):171–9. doi: 10.9758/cpn.2014.12.3.171 ; PubMed Central PMCID: PMC4293161. 25598819 PMC4293161

[84] Haidar SA, de Vries NK, Karavetian M, El-Rassi R. Stress, Anxiety, and Weight Gain among University and College Students: A Systematic Review. J Acad Nutr Diet. 2018;118(2):261–74. doi: 10.1016/j.jand.2017.10.015 . 29389509

[85] Yang C, Chen A, Chen Y. College students’ stress and health in the COVID-19 pandemic: The role of academic workload, separation from school, and fears of contagion. PLoS One. 2021;16(2):e0246676. doi: 10.1371/journal.pone.0246676 ; PubMed Central PMCID: PMC7875391. 33566824 PMC7875391

[86] World Health Organization. The ICD-10 classification of mental and behavioural disorders: diagnostic criteria for research. Available from: https://apps.who.int/iris/handle/10665/37108 1993.

[87] Lupien SJ, McEwen BS, Gunnar MR, Heim C. Effects of stress throughout the lifespan on the brain, behaviour and cognition. Nat Rev Neurosci. 2009;10(6):434–45. doi: 10.1038/nrn2639 . 19401723

[88] Staniloiu A, Markowitsch HJ. Towards solving the riddle of forgetting in functional amnesia: recent advances and current opinions. Front Psychol. 2012;3:403. doi: 10.3389/fpsyg.2012.00403 ; PubMed Central PMCID: PMC3485580. 23125838 PMC3485580

[89] Labonte B, Suderman M, Maussion G, Navaro L, Yerko V, Mahar I, et al. Genome-wide epigenetic regulation by early-life trauma. Arch Gen Psychiatry. 2012;69(7):722–31. doi: 10.1001/archgenpsychiatry.2011.2287 ; PubMed Central PMCID: PMC4991944. 22752237 PMC4991944

[90] Heim CM, Mayberg HS, Mletzko T, Nemeroff CB, Pruessner JC. Decreased cortical representation of genital somatosensory field after childhood sexual abuse. Am J Psychiatry. 2013;170(6):616–23. doi: 10.1176/appi.ajp.2013.12070950 . 23732967

[91] Soffer-Dudek N. Daily Elevations in Dissociative Absorption and Depersonalization in a Nonclinical Sample Are Related to Daily Stress and Psychopathological Symptoms. Psychiatry. 2017;80(3):265–78. doi: 10.1080/00332747.2016.1247622 . 29087250

[92] Giesbrecht T, Smeets T, Merckelbach H, Jelicic M. Depersonalization experiences in undergraduates are related to heightened stress cortisol responses. J Nerv Ment Dis. 2007;195(4):282–7. doi: 10.1097/01.nmd.0000253822.60618.60 . 17435477

[93] Jauregui Renaud K, Cooper-Bribiesca D, Martinez-Pichardo E, Miguel Puga JA, Rascon-Martinez DM, Sanchez Hurtado LA, et al. Acute Stress in Health Workers during Two Consecutive Epidemic Waves of COVID-19. Int J Environ Res Public Health. 2021;19(1). doi: 10.3390/ijerph19010206 ; PubMed Central PMCID: PMC8751091. 35010465 PMC8751091

[94] Sacco RG. The circumplex structure of depersonalization/derealization. International Journal of Psychological Studies. 2010;2(2):26.

[95] Simeon D, Abugel J. Feeling unreal: Depersonalization disorder and the loss of the self: Oxford University Press, USA; 2006.

[96] Morgan CA 3rd, Southwick S, Hazlett G, Rasmusson A, Hoyt G, Zimolo Z, et al. Relationships among plasma dehydroepiandrosterone sulfate and cortisol levels, symptoms of dissociation, and objective performance in humans exposed to acute stress. Arch Gen Psychiatry. 2004;61(8):819–25. doi: 10.1001/archpsyc.61.8.819 . 15289280

[97] Rodewald F, Dell PF, Wilhelm-Gossling C, Gast U. Are major dissociative disorders characterized by a qualitatively different kind of dissociation? J Trauma Dissociation. 2011;12(1):9–24. doi: 10.1080/15299732.2010.514847 . 21240735

[98] Lemche E, Surguladze SA, Brammer MJ, Phillips ML, Sierra M, David AS, et al. Dissociable brain correlates for depression, anxiety, dissociation, and somatization in depersonalization-derealization disorder. CNS Spectr. 2016;21(1):35–42. doi: 10.1017/S1092852913000588 . 24059962

[99] Mula M, Pini S, Cassano GB. The neurobiology and clinical significance of depersonalization in mood and anxiety disorders: a critical reappraisal. J Affect Disord. 2007;99(1–3):91–9. doi: 10.1016/j.jad.2006.08.025 . 16997382

[100] Schlax J, Wiltink J, Beutel ME, Munzel T, Pfeiffer N, Wild P, et al. Symptoms of depersonalization/derealization are independent risk factors for the development or persistence of psychological distress in the general population: Results from the Gutenberg health study. J Affect Disord. 2020;273:41–7. doi: 10.1016/j.jad.2020.04.018 . 32421621

